# Reduced Efficacy of Piperonyl Butoxide Based Long-Lasting Insecticide-Treated Nets Against Malaria Vectors in Western Kenya

**DOI:** 10.4269/ajtmh.25-0342

**Published:** 2026-04-25

**Authors:** Samuel Kahindi, Lucy Abel, Erastus Kirwa, Mark Amunga, Emma Kimachas, Evans Omondi, Millicent Cherono, Judith Mangeni, Andrew Obala, Christine Markwalter, Wendy Prudhomme O’Meara

**Affiliations:** ^1^ School of Pure and Applied Sciences, Pwani University, Kilifi, Kenya;; ^2^ Academic Model Providing Access to Health Care (AMPATH), Eldoret, Kenya;; ^3^ School of Public Health, College of Health Sciences, Moi University, Eldoret, Kenya;; ^4^ School of Medicine, College of Health Sciences, Moi University, Eldoret, Kenya;; ^5^ Duke Global Health Institute, Duke University, Durham, North Carolina, USA

## Abstract

In response to rising insecticide resistance among malaria vector species, countries are implementing long-lasting insecticide treated nets (LLINs) coformulated with piperonyl butoxide (PBO), which can restore susceptibility to pyrethroid-based nets. In mid-2021, Kenya National Malaria Program rolled out PBO-LLINs in Bungoma County. We evaluated the susceptibility of wild-collected vectors to new versus used PBO-LLINs using the WHO cone bioassays. Results were confirmed with the WHO susceptibility assay. A total of 2,020 female *Anopheles* were tested, of which 1,480 were wild-collected and 540 were a Kilifi-susceptible strain. Mortality after exposure to new PBO-LLINs was only 68% in the wild-collected mosquitoes and as low as 34% when mosquitoes were exposed to used, 24-month old PBO-LLINs. *Anopheles arabiensis* was the main vector species that survived exposure. Susceptibility tests confirmed very low mortality after exposure to permethrin with or without PBO. Reduced sensitivity can be attributed partly to the aging of the net but also to very high levels of resistance in the local vector population.

## INTRODUCTION

The introduction of long-lasting insecticide treated bed nets (LLINs) has been hailed for lowering malaria transmission in endemic areas by killing foraging mosquitoes as they come in contact with the net.^1^^,^^2^ However, concerns about net efficacy in the face of increasing resistance to pyrethroids, the primary class of insecticides used in LLINs, have been raised.^3^^–^^5^ Next-generation LLINs are formulated with multiple compounds to improve efficacy and reduce the spread of insecticide resistance. One such product is the coformulation of LLINs with piperonyl butoxide (PBO) which acts as a synergist and restores sensitivity of resistant insects to pyrethroids.^6^^,^^7^ PBO nets are particularly effective in areas where mosquitoes have developed resistance to standard pyrethroid insecticides^8^ demonstrating higher mortality rates in resistant mosquito populations^6^^,^^9^ in comparison with standard pyrethroid-only nets. Various field trials in Africa, including Kenya, Uganda and Tanzania, have demonstrated that PBO-LLINs significantly reduce malaria incidence compared with standard LLINs.^9^^–^^12^ Although PBO-LLINs are effective for restoring the efficacy of pyrethroid-based nets in field trials,^8^ the effectiveness and duration of protection under programmatic settings has not been established.^13^ In mid-2021, the Kenya National Malaria Program rolled out PBO-LLINs in two counties, including Bungoma County, to combat rising malaria cases likely attributable in part to vector sensitivity to pyrethroid-based nets. We evaluated the efficacy of the Olyset™ Plus PBO-LLINs against wild-collected malaria vectors across four villages in Bungoma County, 2 years after distribution.

## MATERIALS AND METHODS

The study was carried out in Bungoma county,^14^ where malaria transmission is moderate^15^ and perennial. Olyset Plus nets (Sumitomo Chemical, New York, NY) containing permethrin (20 g/kg) and PBO (10 g/kg) were distributed to all households in July–August 2021. PBO-LLINs (Olyset Plus, permethrin 20 g/kg, and PBO 10 g/kg) used in households for 24 months were randomly selected from 11 households in four villages. Untreated nets (100% Polyester, fine mesh and white in color) were used as negative control. New, unused PBO-LLIN (Olyset Plus) was used as a positive control. One new deltamethrin-based and a used permethrin-based conventional (without PBO) LLIN were also tested.

Wild *Anopheles* were collected as larvae from aquatic habitats across the same villages between June 2023 and February 2024. Larvae/pupae were reared using standard techniques until they emerged. Mosquitoes were maintained under standard conditions of temperatures of 27 ± 2°C and 75% ± 10% relative humidity, with a 12-hour day/night cycle. Non–blood-fed, 2–5-day old female *Anopheles* were used for all assays. A fully susceptible, laboratory-reared *An. gambiae* s.s. strain (“Kilifi strain”) was used as a reference.^16^

The nets were tested following the WHO cone bioassay protocol,^17^ where a 30-cm × 30-cm piece was cut from two sides of the net and tested by exposing five female *Anopheles* in a standard cone in four replicates for 3 minutes, after which they were transferred into paper cups.

Susceptibility tests were carried out on the wild-collected *Anopheles* mosquitoes using the standard WHO tube bioassay protocol.^18^ The pyrethroids tested were deltamethrin 0.05%, permethrin 0.75%, and alphacypermethrin 0.05%. Twenty unfed female *Anopheles* were exposed to each insecticide in four replicates by introducing them into tubes with test papers. Similarly, four replicates of 20 mosquitoes were exposed to PBO 4% papers for 60 minutes before pyrethroid exposure to compare the effect of the pyrethroid with and without PBO. Mosquitoes were exposed to untreated papers in two replicates as a control.

For both assays, we recorded the number of mosquitoes knocked down after one hour and killed after 24 hours. Mosquitoes were maintained on 10% sucrose. For the cone assay, surviving and succumbed wild mosquitoes were morphologically identified into species.^19^ Members of the *An. gambiae* complex were further identified as either *An. arabiensis*, *An. gambaie* s.s., or other by polymerase chain reaction (PCR).^20^

## RESULTS

A total of 1,180 female *Anopheles* were tested using cone bioassays; 640 locally caught wild mosquitoes and 540 Kilifi strain. In addition, 380 mosquitoes were exposed to an untreated net, and 800 were exposed to treated nets (Table 1). A total of 15 nets were tested; 11 used PBO-LLINs, 1 new unused PBO-LLIN, 1 used 1 new conventional LLIN, and 1 used an untreated net (negative control).

**Table 1:** Knockdown and mortality in *Anopheles* mosquitoes by PBO and conventional nets

Net Type	Nets (*n*)	Mosquitos (*N*)	Susceptible			Wild		
			Exposed, *n* (%)	Knocked Down, *n* (% ± SE)	Killed, *n* (% ± SE)	Exposed, *n* (%)	Knocked Down, *n* (% ± SE)	Killed, *n* (% ± SE)
Untreated	1	380	180 (47)	0 (0 ± 0)	0 (0 ± 0)	200 (53)	0 (0 ± 0)	0 (0 ± 0)
Pyrethroid only								
New	1	40	20 (50)	20 (100 ± 0)	20 (100 ± 0)	20 (50)	9 (45 ± 10)	5 (25 ± 10)
Used	1	40	20 (50)	18 (90 ± 6)	14 (70 ± 13)	20 (50)	6 (30 ± 13)	4 (20 ± 14)
Pyrethroid plus PBO^†^								
New	1	60	20 (33)	20 (100 ± 0)	20 (100 ± 0)	40 (66)	38 (95 ± 6)	27 (68 ± 3)
Used	11	660	300 (45)	259 (86 ± 2)	287 (96 ± 1)	360 (55)	168 (47 ± 3)	124 (34 ± 3)

PBO = piperonyl butoxide; SE = standard error.

^†^ Olyset plus–permethrin (20 g/kg) and PBO (10 g/kg).

Mortality after exposure to treated nets was significantly lower in the wild-collected versus susceptible vectors. Wild mosquitoes exposed to the unused deltamethrin LLIN had a knockdown of 45 ± 10% (9/20) and mortality of 25 ± 10% (5/20) in comparison with 100% (20/20) knockdown and 100% (20/20) mortality for the fully susceptible Kilifi strain. The unused PBO-LLIN resulted in 95 ± 6% (38/40) knockdown and 68 ± 3% (27/40) mortality among the wild mosquitoes, whereas the knockdown and mortality for the susceptible strain was 100% (20/20). As expected, there was no knockdown or mortality in the control assays with untreated nets (Table 1).

The used conventional LLIN resulted in only 30 ± 13% (6/20) knockdown and 20 ± 14% (4/20) mortality for the wild mosquitoes, in comparison with 90 ± 6% (18/20) knockdown and 70 ± 13% (14/20) mortality in the susceptible strain (Table 1). Used PBO-LLINs gave a knockdown of 47 ± 3% (168/360) and mortality of 34 ± 3% (124/360) for the wild-collected mosquitoes, in comparison with 86 ± 2% (259/300) knockdown and 96 ± 1% (287/300) mortality in the susceptible strain (Table 1).

**Figure 1.**
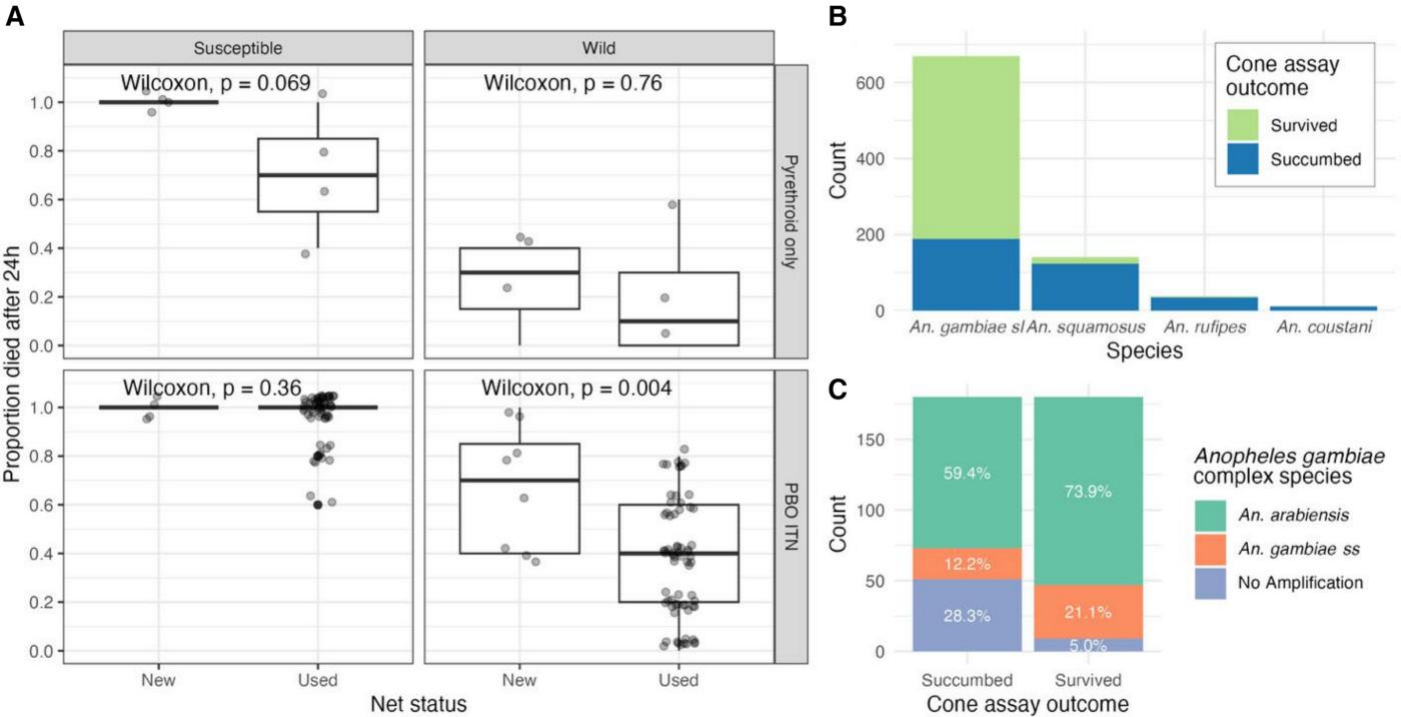
(**A**) *Anopheles* mortality rates for new and used conventional and PBO LLINs across susceptible and wild strains. Points represent individual replicates (*n* = 5 mosquitoes each) and are jittered for visualization. Box plots show medians and interquartile ranges. (**B**) Species distribution of wild *Anopheles* mosquitoes tested by cone assay, identified morphologically. Color indicates whether mosquitoes survived versus succumbed. (**C**) Distribution of members of *Anopheles gambiae* complex mosquitoes, identified by polymerase chain reaction, in the succumbed and survived arms of the cone assay.

There was a significant difference in mortality between the new and used PBO-LLINs for the wild mosquitoes (*P* = 0.004); however, there was no statistical difference in mortality of wild-collected vectors exposed to used and unused pyrethroid-only LLINs (Figure 1). For the susceptible strain, mortality was high for both PBO and conventional nets, and there was no significant difference in mortality between new and used nets of either type.

Morphological identification revealed most of the wild-collected *Anopheles* belonged to the *An. gambiae* complex (77.8%), followed by *An. squamosus* (16.4%) (Figure 1B). Most of the mosquitoes that survived exposure to insecticide belonged to the *An. gambiae* complex (96%). Molecular analysis of a subset of the *An. gambiae* complex (*n* = 180 survived, *n* = 180 succumbed) showed *An. arabiensis* was overrepresented in the sample of mosquitoes that survived in comparison with those that succumbed (Figure 1C).

**Figure 2.**
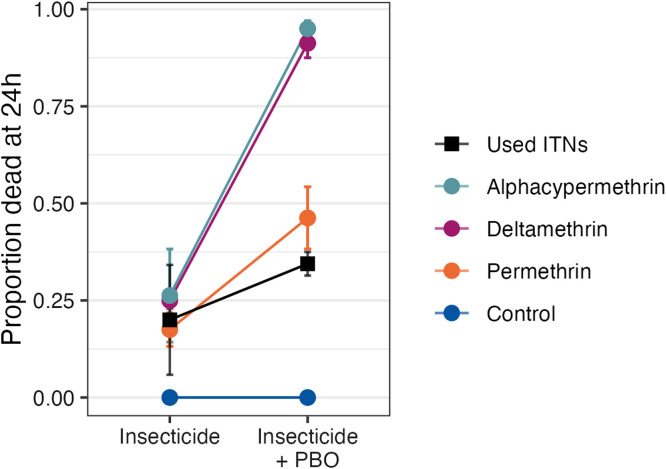
Mortality of wild local vectors when exposed in standard WHO susceptibility assays to a pyrethroid-impregnated paper or exposed to piperonyl butoxide (PBO) followed by a pyrethroid. Control experiments were conducted with plain paper with or without preexposure to PBO paper and show that there is no direct mortality effect of the PBO. The black squares show mortality after exposure to used nets (permethrin-impregnated with and without PBO) in the cone assay for comparison. Points represent mean mortality rates and error bars show the standard error.

To assess resistance, 840 female wild *Anopheles* were tested in susceptibility bioassays; 240 were exposed to untreated papers and 560 to pyrethroid-treated papers. After 24 hours, mortality in wild-collected *Anopheles* due to deltamethrin was 25 ± 2% (20/80), alphacypermethrin at 26 ± 12% (21/80), and permethrin at 18 ± 4% (14/80) (Figure 2). When pyrethroids were tested after exposure to PBO, mortality by deltamethrin plus PBO was 91 ± 4% (73/80), alphacypermethrin plus PBO at 95 ± 2% (76/80), and permethrin plus PBO at 46 ± 8% (37/80). Mortality was 0% (0/80) for the untreated control.

## DISCUSSION

Several studies have reported malaria vector resistance against pyrethroid insecticides used in LLINs. This resistance has been implicated in the reduced efficacy of the LLINs in controlling malaria transmission. Adding the synergist PBO to pyrethroid-based LLINs can restore susceptibility of resistant vectors. This study compared the survival of wild-collected vectors and fully susceptible laboratory strains to new and used LLINs with or without PBO. Insecticide resistance in across the major malaria vectors in western Kenya has been a growing concern, threatening the efficacy of vector control strategies such as the use of LLINs and IRS. Studies indicate that the resistance intensity is significant^22^^–^^24^ and is strongly thought to be due to varying agrochemical use in various agricultural ecosystems.^25^ *An. gambiae* complex, the major malaria vector in Bungoma, Kenya, has been reported to show high levels of resistance toward pyrethroids via the knockdown resistance (kdr) mechanism,^21^ and this could possibly explain why even the addition of PBO in the LLINs could not restore full susceptibility in these vectors.

The PBO-LLINs collected across four villages resulted in very low mortality in the wild *Anopheles* mosquitoes after two years of use. This could be due in part to the fact that the PBO degrades more quickly than the pyrethroid with which it synergizes; similar observations have been reported in other studies,^13^^,^^21^ including a study in Tanzania that reported very low levels of PBO in Olyset Plus nets after 24 months of usage.^9^ However, susceptibility to new PBO-LLIN among wild vectors was less than 70%, suggesting that high levels of resistance plus PBO degradation are both contributing to low effectiveness of the nets.

Susceptibility tests also showed that all the four pyrethroids tested resulted in very low mortalities in the local vectors, below the WHO threshold for high pyrethroid resistance. Mortality was only partially restored with PBO exposure. This finding was particularly striking in the permethrin plus PBO combination, where mortality was <50% of the exposed mosquitoes, suggesting very limited utility of permethrin with or without PBO in this region. These observations are consistent with other studies in Bungoma^22^ and corroborate the observed low mortalities in the net assays.

The increase in mortality when PBO was combined with pyrethroids indicates involvement of P450 monooxygenase as detoxifying enzymes conferring resistance against the pyrethroids. The addition of PBO was able to suppress the activity of the detoxifying enzymes and thus increase the sensitivity of the vectors toward the pyrethroids. However, in the case of permethrin, which demonstrated low mortality in wild-collected vectors without (18 ± 4%) and with (46 ± 8%) PBO, the persistent resistance of the wild vectors even when PBO was present indicates an additional mechanism of resistance other than the P450 monooxygenases.

*An. gambiae* s.l. was the predominant species collected and reared from this region. Molecular analysis showed that *An. arabiensis* was the main species of the complex and the largest portion of the population that survived exposure. A total of 25% of the *An. gambiae* s.l. that succumbed were neither *An. arabiensis* nor *An. gambiae* s.s., and it is unknown whether this subset of mosquitoes is contributing to human biting and malaria transmission.

The WHO recommends 3 years between net distribution campaigns. However, we observed PBO-LLINs have very low efficacy against local malaria vector populations after only 2 years of use. This low efficacy is attributed to net age and to high levels of resistance in the local vector population. The level of protection against malaria infection is likely to be greatly attenuated and short-lived.

This study had some limitations. First, the main focus of the study was to evaluate the efficacy of the used PBO LLINs (Olyset Plus) that were currently in use in the community 2 years after mass distribution. The other test LLIN categories, the pyrethroid-only (both new and used), were just for quick comparison purposes only. In this regard we prioritized using the mosquitoes for testing the PBO-LLINs, and thus very few mosquitoes were tested for the new PBO and pyrethroid-only LLINs. We strongly believe this does not compromise the outcomes on the efficacy of the used PBO-LLINs tested. Second, *An. funestus* mosquitoes were not collected for testing, though this vector is present in the study area but in lower proportion in comparison with the *An. gambiae* complex. This is because field mosquitoes were collected as larvae in the readily available open water pools within the villages, whereas *An. funestus* prefer to breed in habitats with lots of vegetation, especially rivers and streams where larval collection is in most cases not very practical and also does not yield high numbers of larvae ideal for bioassays.

## References

[1] Barreaux P Ranson H Foster GM McCall PJ, 2023. Pyrethroid-treated bed nets impair blood feeding performance in insecticide resistant mosquitoes. Sci Rep 13(1): 10055. 37344580 10.1038/s41598-023-35958-z PMC10284836

[2] Miller JE Lindsay SW Armstrong JR, 1991. Experimental hut trials of bednets impregnated with synthetic pyrethroid or organophosphate insecticide for mosquito control in The Gambia. Med Vet Entomol 5(4): 465–476. 1685337 10.1111/j.1365-2915.1991.tb00575.x

[3] Lindblade KA, , 2015. A cohort study of the effectiveness of insecticide-treated bed nets to prevent malaria in an area of moderate pyrethroid resistance, Malawi. Malar J 14(1): 31. 25627987 10.1186/s12936-015-0554-1 PMC4318190

[4] Lindsay SW Thomas MB Kleinschmidt I, 2021. Threats to the effectiveness of insecticide-treated bednets for malaria control: Thinking beyond insecticide resistance. Lancet Glob Health 9(9): e1325–e1331. 34216565 10.1016/S2214-109X(21)00216-3

[5] N’Guessan R Corbel V Akogbéto M Rowland M, 2007. Reduced efficacy of insecticide-treated nets and indoor residual spraying for malaria control in pyrethroid resistance area, Benin. Emerg Infect Dis 13(2): 199–206. 17479880 10.3201/eid1302.060631 PMC2725864

[6] Kariuki S Kamau L, 2022. A new generation of long-lasting insecticidal nets. Lancet 399(10331): 1202–1203. 35339212 10.1016/S0140-6736(22)00004-6

[7] Messenger LA, , 2023. Effects of next-generation, dual-active-ingredient, long-lasting insecticidal net deployment on insecticide resistance in malaria vectors in Tanzania: An analysis of a 3-year, cluster-randomised controlled trial. Lancet Planet Health 7(8): e673–e683. 37558348 10.1016/S2542-5196(23)00137-7

[8] Allossogbe M Gnanguenon V Yovogan B Akinro B Anagonou R Agossa F Houtoukpe A Padonou GG Akogbeto M, 2017. WHO cone bio-assays of classical and new-generation long-lasting insecticidal nets call for innovative insecticides targeting the knock-down resistance mechanism in Benin. Malar J 16(1): 77. 28202024 10.1186/s12936-017-1727-x PMC5312429

[9] Protopopoff N, , 2023. Effectiveness of piperonyl butoxide and pyrethroid-treated long-lasting insecticidal nets (LLINs) versus pyrethroid-only LLINs with and without indoor residual spray against malaria infection: Third year results of a cluster, randomised controlled, two-by-two factorial design trial in Tanzania. Malar J 22(1): 294. 37789389 10.1186/s12936-023-04727-8 PMC10548685

[10] Gichuki PM Kamau L Njagi K Karoki S Muigai N Matoke-Muhia D Bayoh N Mathenge E Yadav RS, 2021. Bioefficacy and durability of Olyset^®^ Plus, a permethrin and piperonyl butoxide-treated insecticidal net in a 3-year long trial in Kenya. Infect Dis Poverty 10(1): 135. 34930459 10.1186/s40249-021-00916-2 PMC8691082

[11] Martin JL Mosha FW Lukole E Rowland M Todd J Charlwood JD Mosha JF Protopopoff N, 2021. Personal protection with PBO-pyrethroid synergist-treated nets after 2 years of household use against pyrethroid-resistant *Anopheles* in Tanzania. Parasit Vectors 14(1): 150. 33691742 10.1186/s13071-021-04641-5 PMC7944899

[12] Gonahasa S, , 2025. LLIN Evaluation in Uganda Project (LLINEUP2) – Effect of long-lasting insecticidal nets (LLINs) treated with pyrethroid plus pyriproxyfen vs LLINs treated with pyrethroid plus piperonyl butoxide in Uganda: A cluster-randomised trial. PLoS Glob Public Health 5(2): e0003558. 40009611 10.1371/journal.pgph.0003558 PMC11864545

[13] Nkahe DL Kopya E Ngangue Siewe NI Ndjeunia Mbiakop P Kala Chouakeu NA Mimpfoundi R Kekeunou S Awono-Ambene P Antonio-Nkondjio C, 2024. Durability of PBO nets (Olyset Plus^®^), 12 months after their distribution in Bertoua, Cameroon. Parasite Epidemiol Control 26: e00373. 39228793 10.1016/j.parepi.2024.e00373 PMC11369369

[14] Abel L, , 2024. Relationship between malaria vector survival, infectivity, and insecticide-treated net use in western Kenya. Parasit Vectors 17(1): 464. 39533350 10.1186/s13071-024-06550-9 PMC11558830

[15] Sumner KM Freedman E Abel L Obala A Pence BW Wesolowski A Meshnick SR Prudhomme-O’Meara W Taylor SM, 2021. Genotyping cognate *Plasmodium falciparum* in humans and mosquitoes to estimate onward transmission of asymptomatic infections. Nat Commun 12(1): 909. 33568678 10.1038/s41467-021-21269-2 PMC7875998

[16] Kiuru C Ominde K Muturi M Babu L Wanjiku C Chaccour C Maia MF, 2023. Effects of larval exposure to sublethal doses of ivermectin on adult fitness and susceptibility to ivermectin in *Anopheles gambiae* s.s. Parasit Vectors 16(1): 293. 37605264 10.1186/s13071-023-05888-w PMC10441747

[17] World Health Organization, WHO Pesticide Evaluation Scheme, 2013. *Guidelines for Laboratory and Field-Testing of Long-Lasting Insecticidal Nets*. Available at: https://iris.who.int/handle/10665/80270. Accessed April 6, 2026.

[18] World Health Organization. *Manual for Monitoring Insecticide Resistance in Mosquito Vectors and Selecting Appropriate Interventions*. Available at: www.who.int/publications/i/item/9789240051089. Accessed August 12, 2025.

[19] Coetzee M, 2020. Key to the females of Afrotropical *Anopheles* mosquitoes (Diptera: Culicidae). Malar J 19(1): 70. 32054502 10.1186/s12936-020-3144-9 PMC7020601

[20] Wilkins EE Howell PI Benedict MQ, 2006. IMP PCR primers detect single nucleotide polymorphisms for *Anopheles gambiae* species identification, Mopti and Savanna rDNA types, and resistance to dieldrin in *Anopheles arabiensis*. Malar J 5: 125. 17177993 10.1186/1475-2875-5-125 PMC1769388

[21] Gleave K Lissenden N Chaplin M Choi L Ranson H, 2021. Piperonyl butoxide (PBO) combined with pyrethroids in insecticide-treated nets to prevent malaria in Africa. Cochrane Database Syst Rev 5(5): CD012776. 34027998 10.1002/14651858.CD012776.pub3 PMC8142305

[22] Omoke D Kipsum M Otieno S Esalimba E Sheth M Lenhart A Njeru EM Ochomo E Dada N, 2021. Western Kenyan *Anopheles gambiae* showing intense permethrin resistance harbour distinct microbiota. Malar J 20(1): 77. 33557825 10.1186/s12936-021-03606-4 PMC7869237

[23] Machani MG, , 2024. Insecticide resistance and its intensity in urban *Anopheles arabiensis* in Kisumu City, Western Kenya: Implications for malaria control in urban areas. PLoS One 19(11): e0303921. 39536003 10.1371/journal.pone.0303921 PMC11560014

[24] Wanjala CL Kweka EJ, 2018. Malaria vectors insecticides resistance in different agroecosystems in western Kenya. Front Public Health 6: 55. 29546039 10.3389/fpubh.2018.00055 PMC5838019

[25] Orondo P, , 2021. Insecticide resistance status of *Anopheles arabiensis* in irrigated and non-irrigated areas in western Kenya. Parasit Vectors 14(1): 335. 34174946 10.1186/s13071-021-04833-z PMC8235622

